# Using simultaneous equation modeling for defining complex phenotypes

**DOI:** 10.1186/1471-2156-4-S1-S10

**Published:** 2003-12-31

**Authors:** Terri M King

**Affiliations:** 1 Department of Internal Medicine, Division of Medical Genetics, The University of Texas – Houston Medical School, 6431 Fannin Street, Houston, Texas, USA

## Abstract

**Background:**

Interactions between multiple biological phenotypes are difficult to model. Simultaneous equation modelling (SEM), as used in econometric modelling, may prove an effective tool for this problem. Generalized linear models were used to derive the structural equations defining the interactions between cholesterol, glucose, triglycerides and high-density lipoprotein cholesterol (HDL-C). These structural equations were then applied, using SEM, to Cohort 2 data (replicates 1–100) to estimate the phenotypic structure underlying the simulation. The goal was to determine if this empiric method of deriving structural equations for use in SEM was able to recover the simulation model better than generalized linear models.

**Results:**

First, the underlying structural equations were estimated using generalized linear model techniques, which found strong a relationship between glucose, triglycerides and HDL-C. Using these structural equations, I used SEM to evaluate these relationships jointly. I found that a combination of the empiric structural equations and the SEM method was better at recovering the underlying simulated relationship between biologic measures than generalized linear modelling.

**Conclusion:**

The empiric SEM procedure presented here estimated different relationships between dependent variables than generalized linear modelling. The SEM procedure using empirically developed structural equations was able to recover the underlying simulation relationship partially and thus holds promise as a technique for complex phenotype analysis. Robust methods for determining the structural equations must be developed for application of SEM to population data.

## Background

To investigate complex relationships of interrelated phenotypes, I investigated whether simultaneous equation modelling (SEM) techniques can be used to detect this relationship in the absence of knowledge about the system. Simultaneous equation models describe two or more structural equations in which the dependent variable in one equation is a predictive variable in another. A classic example from econometrics is the description of supply and demand within a population [1].

SEMs are attractive in longitudinal genetic studies because they have the ability to include 1) fixed data (e.g., genotypes), 2) variable data (e.g., cholesterol), and 3) stochastic data (e.g., cohort data) [2]. Using Problem Set 2 (complete data, replicates 1–100) without knowledge of the simulation structure, I examined a method to derive empirically the structural equations used in SEM to model the interrelationship phenotype at the first measurement point. The focus of this paper is to compare the ability to recover the simulation structure of traditional generalized linear models (GLM) to empirically derived structural equations used in SEM. This modelling technique would be useful in removing nongenetic components of variance prior to mapping efforts.

## Results

### Deriving structural equations

A summary of the GLM results is presented in Table 1. Using the results of the GLM analyses, the following structural equations were derived:

*Cholesterol *= *c*_1 _+ α_1 _(*age*) + α (*spb*) + α_3 _(*sex*) + *U*_1 _    (1)

*Glucose *= *c*_2 _+ α_4 _(*HDL-C*) +α_5 _(*sex*) + α_6 _(*trig*) + α_7 _(*wgt*) + *U*_2 _    (2)

*HDL-C *= *c*_3 _+ α_8 _(*age*) + α_9 _(*cpd*) + α_11 _(*gluc*) + α_12 _(*sex*) + α_13 _(*trig*) + α_14 _(*wgt*) + *U*_3 _    (3)

*Trig *= *c*_4 _+ α_15 _(*age*) + α_16 _(*cpd*) + α_17 _(*drink*) + α_18 _(*gluc*) + α_19 _(*HDL-C*) + α_20 _(*hgt*) + α_21 _(*sex*) + α_22 _(*wgt*) + *U*_4 _    (4)

These results indicate the cholesterol is not a component of this system of structural models and thus was not included in the SEM analysis.

### Estimation of SEMs

Table 2 presents the results from the SEMs. The significant predictors of glucose in this system were: alcohol consumption, triglycerides, and weight. There were no significant predictors of high-density lipoprotein cholesterol (HDL-C). Finally, the significant predictors of triglycerides were: alcohol consumption, glucose, and weight. The direct generating variables in the simulation equations are denoted in italics in Table 2.

## Discussion

Fitting the GLMs consistently included more covariates than were used in the actual simulation equations. However, when the linear models were used to screen variable for structural equations and then SEM were used to determine the system, I was more successful in defining the underlying system.

The research presented here does not adequately address a number of key features that must be evaluated before endorsing this method. These include detection of nonlinear and higher order relationships, the appropriate detection and adjustment of the correlation structure within the covariates, and estimation procedures in nonreplicate data. However, despite these elements being excluded from this research, I was encouraged by the ability of this method to provide a closer approximation to the simulation system than did GLMs.

## Conclusions

These results suggest that SEM can provide an alternative way to recover unknown relationships in complex phenotype data. The method presented here may result in a reduction in the model parameters that is overly conservative. Factors that must be evaluated in this relationship include the impact of the degree of correlation between the dependent and independent variables and the ability to detect a relationship with SEM.

This data structure seemed ideal to explore the usefulness of simultaneous equations for detailed deconstruction of complex phenotypes. This methodology, however, will need to overcome the challenges of defining robust structural models in the absence of knowledge of the underlying system. In this simulation study, I had the advantage of a large number of replicates, which, in real data, does not exist. I am currently investigating additional methods for determining the structural equations in undefined systems.

## Methods

### Data

Cohort 2 from replicates 1–100 was used in the complete data set without knowledge of the simulation conditions. The structural models were developed around four phenotypes at the first measurement time: cholesterol, glucose, HDL-C, and triglycerides, primarily because of literature focusing on the interrelationship of these agents [3,4]. Covariates included sex, age, height (hgt), systolic blood pressure (sbp), cigarettes per day (cpd), alcohol consumption (drink), and weight (wt). Data were evaluated independent of familial structure. Covariates were tested and found to be normally distributed.

### Identification of the linear systems

The first step was to determine the structural equations that would be used in this analysis. To establish the structural equations, GLMs were fit in each of the 100 replicates to determine which of the covariates was significantly associated with each of the phenotypes. Using Proc GLM, within each replicate, the four phenotypes were analyzed with the following model structure.

*phenotype*_*a *_= *intercept *+ *phenotype*_*b *_+ *phenotype*_*c *_+ *phenotype*_*d *_+ *age *+ *cpd *+ *chol *+ *drink *+ *htg *+ *sbp *+ *sex *+ *wgt *    (5)

Over the 100 replicates, the following information was collected on the regression coefficients: number of replicates in which the regression coefficient was significant (*p *< 0.05), the average of regression coefficient, and whether the distribution of the regression coefficient was normally distributed. To establish the structural equations, covariates were selected that had regression coefficients that were significant in more than 25% of the replicates. It is important to note that I was not interested in the value of the regression coefficient *per se*, but rather if that regression coefficient was significant in a percentage of GLM models.

### Estimation of equations

Using equations (1–4) above, the associated parameters (α_4 _- α_22_) were estimated using Proc Syslin within SAS [5] for each replicate. For this analysis, the parameters were estimated using two-stage least-squares techniques, which allow for these violations. In these techniques, the models are restructured with temporary dependent variables that are not in violation of the recursivity assumption. Then the models are estimated using ordinary least-square methods. These results are presented in Table 2.

## Figures and Tables

**Table 1:** Results of Generalized Linear Models to Determine Structural Equations

		Number of Replicates where the regression coefficient was:			
		
Dependent Variable	Independent Variable	Significant	Not Significant	Normally Distributed?	Mean
Cholesterol	**Age^A^**	**100**	**0**	**Yes**	**0.728**
	Cigarettes per day	2	98	Yes	-0.012
	Alcohol consumption	13	87	Yes	0.004
	Glucose	7	93	Yes	0.014
	HDL	10	90	Yes	-0.025
	Height	13	87	Yes	0.008
	**Systolic blood pressure**	**41**	**59**	**Yes**	**0.124**
	**Sex**	**43**	**57**	**Yes**	**3.916**
	Triglycerides	18	82	Yes	0.020
	Weight	11	89	Yes	0.007

Glucose	Age	14	86	Yes	-0.005
	Cigarettes per day	7	93	Yes	0.001
	Cholesterol	15	85	Yes	-0.016
	**Alcohol consumption**	**100**	**0**	**Yes**	**-0.081**
	**HDL**	**95**	**5**	**Yes**	**0.096**
	Height	5	95	Yes	-0.007
	Systolic blood pressure	22	78	Yes	0.020
	**Sex**	**90**	**10**	**Yes**	**2.075**
	**Triglycerides**	**100**	**0**	**No**	**0.081**
	**Weight**	**90**	**10**	**Yes**	**-0.002**

HDL-C	**Age**	**100**	**0**	**Yes**	**0.019**
	Cigarettes per day	10	90	Yes	-0.003
	**Cholesterol**	**100**	**0**	**Yes**	**0.160**
	**Alcohol consumption**	**100**	**0**	**No**	**0.249**
	**Glucose**	**95**	**5**	**Yes**	**0.111**
	Height	9	91	Yes	0.011
	Systolic blood pressure	10	90	Yes	-0.003
	**Sex**	**100**	**0**	**Yes**	**6.058**
	**Triglycerides**	**100**	**0**	**Yes**	**-0.194**
	**Weight**	**100**	**0**	**Yes**	**0.047**

Tryglycerides	**Age**	**100**	**0**	**Yes**	**1.026**
	Cigarettes per day	18	82	Yes	0.012
	**Cholesterol**	**97**	**3**	**Yes**	**0.161**
	**Alcohol consumption**	**100**	**0**	**Yes**	**0.997**
	**Glucose**	**100**	**0**	**Yes**	**0.573**
	**HDL**	**100**	**0**	**Yes**	**-1.163**
	Height	8	92	Yes	0.018
	Systolic blood pressure	17	83	Yes	-0.002
	**Sex**	**100**	**0**	**No**	**-17.819**
	**Weight**	**100**	**0**	**Yes**	**0.268**

^A ^Bolded variables were included in the structural equation.

**Table 2:** Results of the Simultaneous Equations Model

		Summary Statistics of the Regression Coefficients			
		
Dependent Variable	Independent Variable	Mean	Std Dev	Lower CI	Upper CI
Glucose	**Alcohol consumption^A^**	**1.159**	**0.109**	**0.946**	**1.373**
	HDL	0.038	0.040	-0.040	0.116
	Sex	-0.196	0.110	-0.411	0.019
	**Triglycerides**	**-0.311**	**0.034**	**-0.378**	**-0.243**
	*Weight*^B^	***0.276***	***0.009***	***0.258***	***0.294***

HDL	Age	-0.011	0.380	-0.755	0.734
	*Alcohol consumption*	*-5.436*	*186.730*	*-371.427*	*360.555*
	Glucose	2.408	176.659	-343.843	348.659
	Sex	2.470	21.777	-40.212	45.153
	Triglycerides	1.215	51.391	-99.512	101.942
	Weight	-0.384	50.526	-99.414	98.647

Tryglycerides	Age	-0.005	0.043	-0.089	0.078
	***Alcohol consumption***	***3.731***	***0.126***	***3.485***	***3.978***
	***Glucose***	***-3.176***	***0.433***	***-4.025***	***-2.327***
	*HDL*	*0.138*	*0.143*	*-0.142*	*0.418*
	Sex	-0.661	0.653	-1.940	0.619
	***Weight***	***0.877***	***0.114***	***0.563***	***1.285***

^A^Bolded variables were included in the structural equation. ^B^Italicized variables were direct generators in the simulation model.
